# Genetic Target Modulation Employing CRISPR/Cas9 Identifies Glyoxalase 1 as a Novel Molecular Determinant of Invasion and Metastasis in A375 Human Malignant Melanoma Cells In Vitro and In Vivo

**DOI:** 10.3390/cancers12061369

**Published:** 2020-05-26

**Authors:** Jana Jandova, Jessica Perer, Anh Hua, Jeremy A. Snell, Georg T. Wondrak

**Affiliations:** Department of Pharmacology and Toxicology, College of Pharmacy & UA Cancer Center, University of Arizona, Tucson, AZ 85724, USA; jjandova@arizona.edu (J.J.); jperer@arizona.edu (J.P.); anhhua@arizona.edu (A.H.); jeremysnell@email.arizona.edu (J.A.S.)

**Keywords:** malignant melanoma, glyoxalase 1, epithelial–mesenchymal transition, matrix metalloproteinase 9, SCID mouse metastasis model, transwell invasion, CRISPR/Cas9, CMV-driven re-expression

## Abstract

Metabolic reprogramming is a molecular hallmark of cancer. Recently, we have reported the overexpression of glyoxalase 1 (encoded by *GLO1*), a glutathione-dependent enzyme involved in detoxification of the reactive glycolytic byproduct methylglyoxal, in human malignant melanoma cell culture models and clinical samples. However, the specific role of *GLO1* in melanomagenesis remains largely unexplored. Here, using genetic target modulation, we report the identification of *GLO1* as a novel molecular determinant of invasion and metastasis in malignant melanoma. First, A375 human malignant melanoma cells with *GLO1* deletion (A375-*GLO1*_KO) were engineered using CRISPR/Cas9, and genetic rescue clones were generated by stable transfection of KO clones employing a CMV-driven *GLO1* construct (A375-*GLO1*_R). After confirming *GLO1* target modulation at the mRNA and protein levels (RT-qPCR, immunodetection, enzymatic activity), phenotypic characterization indicated that deletion of *GLO1* does not impact proliferative capacity while causing significant sensitization to methylglyoxal-, chemotherapy-, and starvation-induced cytotoxic stress. Employing differential gene expression array analysis (A375-*GLO1*_KO versus A375-*GLO1*_WT), pronounced modulation of epithelial mesenchymal transition (EMT)-related genes [upregulated: *CDH1*, *OCLN*, *IL1RN, PDGFRB, SNAI3*; (downregulated): *BMP1, CDH2, CTNNB1, FN1, FTH1, FZD7, MELTF, MMP2, MMP9, MYC, PTGS2, SNAI2, TFRC, TWIST1, VIM, WNT5A, ZEB1,* and *ZEB2* (up to tenfold; *p* < 0.05)] was observed—all of which are consistent with EMT suppression as a result of *GLO1* deletion. Importantly, these expression changes were largely reversed upon genetic rescue employing A375-*GLO1*_R cells. Differential expression of *MMP9* as a function of *GLO1* status was further substantiated by enzymatic activity and ELISA analysis; phenotypic assessment revealed the pronounced attenuation of morphological potential, transwell migration, and matrigel 3D-invasion capacity displayed by A375-*GLO1*_KO cells, reversed again in genetic rescue clones. Strikingly, in a SCID mouse metastasis model, lung tumor burden imposed by A375-*GLO1*_KO cells was strongly attenuated as compared to A375-*GLO1*_WT cells. Taken together, these prototype data provide evidence in support of a novel function of *GLO1* in melanoma cell invasiveness and metastasis, and ongoing investigations explore the function and therapeutic potential of *GLO1* as a novel melanoma target.

## 1. Introduction

Glyoxalase 1 (encoded by *GLO1;* NM_006708) is a glutathione-dependent enzyme involved in the detoxification of the reactive glycolytic byproduct methylglyoxal (by catalyzing the formation of S-lactoyl-glutathione from methylglyoxal and reduced glutathione) [1,2]. Recent interest has focused on the emerging role of methylglyoxal and (R)-S-Lactoylglutathione as cellular oncometabolites, involved in tumorigenesis-associated metabolic reprogramming, redox dysregulation, and epigenetic recoding that occurs as a result of posttranslational adduction targeting specific proteins including histones [3,4,5,6,7]. Further, a role of *GLO1* in cancer cell chemoresistance has been demonstrated, and the development of pharmacological and genetic strategies modulating *GLO1* for experimental cancer therapy has attracted significant attention [8,9,10,11,12].

Melanoma, a malignant tumor originating from neural crest-derived melanocytes, causes the majority of skin cancer-related deaths. Despite recent progress in targeted therapies, an urgent need exists for the development of novel melanoma-directed molecular strategies [13,14,15]. Recently, we have published our observation that *GLO1* is overexpressed in human malignant melanoma, detectable in cell culture models and patient samples [16]. Numerous studies now support a causative role of *GLO1* dysregulation in various malignancies including those of the breast, colon, liver, lung, prostate, skin, stomach, and thyroid, among many others [4,9,11,17,18,19]. In addition, *GLO1* expression has now been identified as a novel prognostic marker in human gastric cancer patients [20]. Consistent with a role in metabolic reprogramming, as commonly observed in cancer, a significant body of published evidence indicates that *GLO1* expression plays an essential role in maintaining high glycolytic flux (as it occurs in tumors in the context of aerobic glycolysis, commonly referred to as ‘the Warburg effect’), enabling escape from apoptosis, and also facilitating tumorigenic adaptations to hypoxia [5,6].

Recent interest has focused on metabolic rewiring in melanomagenesis, and BRAF^V600E^-driven oncometabolic adaptation is now recognized as an important driver of hyperproliferation and metastasis that also plays a role in the origin of patient resistance to BRAF kinase inhibitor therapy [21,22]. However, in spite of the emerging role of the glyoxalase system in tumorigenesis, the specific role of dysregulated *GLO1* expression in melanomagenesis has remained elusive. Following our earlier research on *GLO1* overexpression observable during melanoma patient progression, we have now employed CRISPR/Cas 9-based *GLO1* deletion and rescue expression, allowing stringent genetic target modulation as examined in A375 human malignant melanoma cells. Here, we report the identification of *GLO1* as a novel molecular determinant of invasion and metastasis in experimental human malignant melanoma observable in vitro and in vivo.

## 2. Results

### 2.1. A375 Human Malignant Melanoma Cells with Genetic GLO1 Deletion (A375-GLO1_KO) Display Sensitization to Methylglyoxal-, Chemotherapy-, and Starvation-Induced Cytotoxic Stress

**Figure 1:**
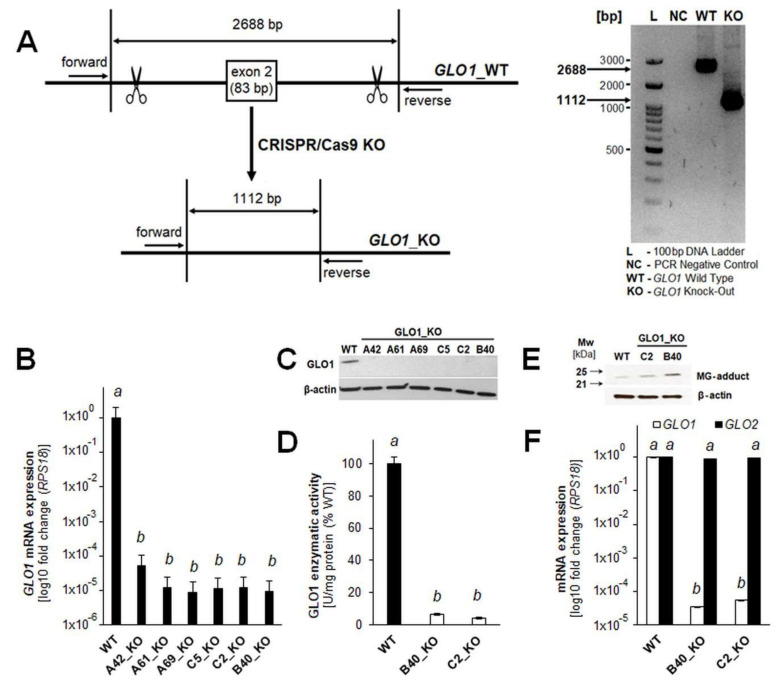
Genomic deletion of *GLO1* in A375 human malignant melanoma cells. (**A**) Exon 2-directed CRISPR/Cas9-dependent *GLO1* deletion (A375-*GLO1*_KO; left panel) was confirmed by PCR analysis of genomic DNA [as compared to wildtype allele (WT; right panel)]. KO status of A375-*GLO1*_KO clones (A42, A61, A69, B40, C2, C5) was validated at the (**B**) mRNA (RT-qPCR) and (**C**) protein (immunoblot analysis) levels. (**D**) Loss of GLO1 enzymatic activity was determined in A375-*GLO1*_KO (B40_KO, C2_KO) clones used for further experimentation. (**E**) Immunoblot detection of methylglyoxal-adducted target proteins using an anti-AGE [arg-pyrimidine] primary antibody. (**F**) The A375-*GLO1*_KO genotype (B40, C2 clones) has no effect on expression levels of *GLO2* mRNA (RT-qPCR; housekeeping gene: *RPS18*). The uncropped blots and molecular weight markers of Figure 1C,E are shown in Figures S1 and S2. For bar graph statistical analysis with letter designation see Materials and Methods Section 4.19.

In order to rigorously test the role of *GLO1* in experimental melanomagenesis, a genetic target modulation approach was pursued (Figure 1). To this end, A375 human melanoma cells, used widely as a cell culture model representative of the BRAF^V600E^-driven malignancy, were chosen to generate clones with *GLO1* deletion (A375-*GLO1*_KO: A42, A61, A69, B40, C2, C5). To this end, a CRISPR/Cas9-mediated exon 2 deletion approach was executed and then confirmed by PCR analysis of genomic DNA (Figure 1A). Successful *GLO1* target modulation was then further substantiated by RT-qPCR and immunodetection, revealing the complete absence of *GLO1* mRNA transcript and protein, respectively, from all analyzed clones as compared to A375-*GLO1*_WT cells (Figure 1B,C). Moreover, consistent with complete obliteration of *GLO1* expression detectable at the mRNA and protein levels, GLO1-specific enzymatic activity was almost completely absent from A375-*GLO1*_KO (B40, C2) clones that were then selected for further experimentation (Figure 1D). Likewise, immunodetection using an antibody directed against the methylglyoxal-specific epitope arg-pyrimidine indicated increased levels of methylglyoxal-adducted target proteins of unknown molecular identity (Figure 1E). We also observed that the A375-*GLO1*_KO genotype (B40, C2) has no effect on expression levels of *GLO2* mRNA as examined by RT-qPCR (Figure 1F).

**Figure 2:**
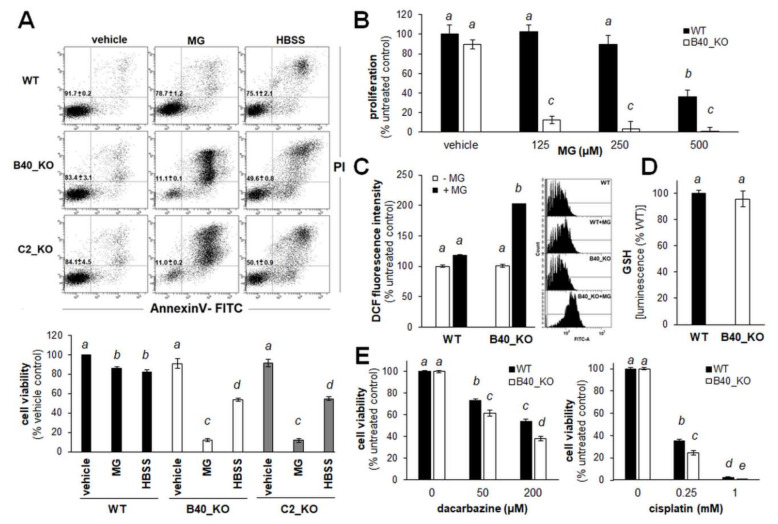
Human A375 malignant melanoma cells with genomic *GLO1* deletion (A375-*GLO1*_KO) display significant sensitization to methylglyoxal (MG)-, chemotherapy-, and starvation-induced cytotoxic stress. (**A**) Impairment of cellular viability (WT, B40_KO, C2_KO) in response to MG (500 µm, 24 h) or serum starvation (HBSS, 24 h) monitored using flow cytometric analysis (annexin V-PI staining). Numbers in quadrants indicate percentage of viable cells (AV negative, PI negative) from a total of gated cells (mean ± SD). Bar graph indicates cell viability as a function of treatment and *GLO1* deletion. (**B**) MG-induced impairment of cellular proliferation (WT, B40_KO; ≤500 µm, 72 h). (**C**) MG-induced oxidative stress (WT, B40_KO; 500 µm, 2 h), as monitored by flow cytometric detection of DCF fluorescence. Left panel: bar graph; right panel: one set of histograms representative of three repeats is shown. (**D**) Intracellular reduced glutathione content (luminescence intensity) normalized to cell number (WT, B40_KO; mean ± SD). (**E**) Impairment of cell viability (WT, B40_KO) in response to dacarbazine (≤200 µm, 24 h; left panel) and cisplatin (≤1 mM, 24 h; right panel). For bar graph statistical analysis with letter designation see Materials and Methods Section 4.19.

Next, phenotypic characterization of selected A375-*GLO1*_KO cells (B40, C2) was performed (Figure 2). As expected, it was observed that genomic depletion of *GLO1* expression sensitizes A375_*GLO1*_KO melanoma cells to methylglyoxal (500 µm; 24 h) cytotoxicity, as assessed by flow cytometric viability analysis of annexinV-PI stained cells (Figure 2A, center panels and bottom bar graph). Likewise, A375_*GLO1*_KO clones displayed increased sensitivity to serum deprivation, observable upon prolonged incubation in serum-free Hank’s balanced salt solution (HBSS), a methodology described by us before (Figure 2A, right panels and bottom bar graph) [23].

Additional phenotypic outcomes of A375_*GLO1*_KO status were examined as follows: Consistent with increased sensitivity to methylglyoxal-induced cell death displayed by A375_*GLO1*_KO cells (Figure 2A), the anti-proliferative effects of methylglyoxal were more pronounced in cells lacking *GLO1* expression (Figure 2B). Likewise, it was observed that methylglyoxal-induced oxidative stress, assessed by flow cytometric analysis of DCF-stained cells, was increased in the absence of *GLO1* expression, a result consistent with earlier observations that methylglyoxal exposure induces cellular oxidative stress antagonized by *GLO1*-dependent detoxification (Figure 2C). However, baseline oxidative stress, as assessed by DCF staining and luminescent determination of the reduced glutathione pool, remained unchanged in *GLO1*_KO clones compared to wildtype cells (Figure 2D). Moreover, since GLO1 enzymatic activity has earlier been described as a molecular factor determining chemoresistance of cancer cells to electrophilic chemotherapeutics, we also assessed the effects of two standard agents (cisplatin and dacarbazine) on viability of A375_*GLO1*_WT versus A375_*GLO1*_KO cells (Figure 2E) [8,24]. A moderate, yet significant increase in sensitivity to cisplatin- and dacarbazine-induced cytotoxicity was observed in cells lacking *GLO1* expression.

Taken together, these data demonstrate that CRISPR/Cas 9-mediated deletion of *GLO1* does not impact proliferative capacity while causing significant sensitization to methylglyoxal-, chemotherapy-, and starvation-induced cytotoxic stress in A375 malignant melanoma cells.

### 2.2. Differential Array Analysis Reveals Pronounced Modulation of EMT-Related Gene Expression in A375-GLO1_KO Cells that Display Attenuated Migration, Invasiveness, and Metastasis Assessed In Vitro and In Vivo

**Figure 3:**
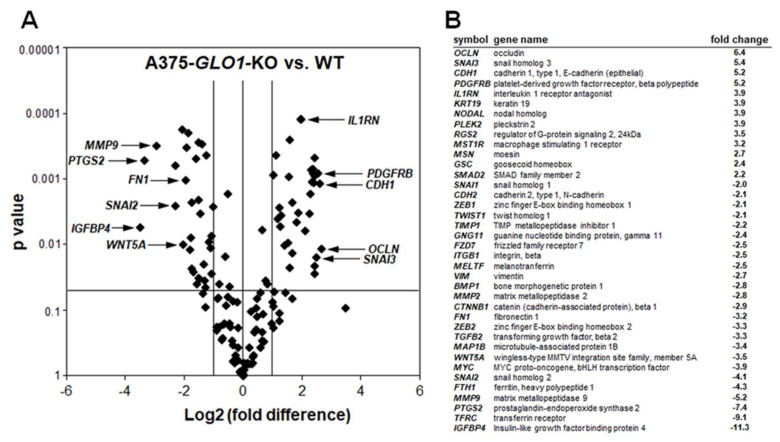
Array analysis indicates downregulation of epithelial mesenchymal transition (EMT)-related gene expression in human A375 malignant melanoma cells with genomic *GLO1* deletion. (**A**) Volcano plot depicting differential gene expression (WT versus B40_KO), as detected by the RT^2^ Profiler^TM^ PCR expression array technology; (cut-off criteria: expression differential > 2; *p* value ≤ 0.05; n = 3). (**B**) Numerical expression changes [B40_KO versus WT] revealing modulation of EMT-related genes as a function of *GLO1* deletion.

In an attempt to further identify tumorigenesis-related changes resulting from genomic *GLO1* deletion, we performed differential gene expression analysis comparing A375-*GLO1*_KO (B40) with A375-*GLO1*_WT cells (Figure 3). Interrogating expression of 168 genes using the RT^2^ Profiler^TM^ PCR expression array technology, significant changes at the mRNA level affecting twenty-one EMT-related genes were detected [(upregulated): *CDH1*, *OCLN*, *IL1RN, PDGFRB, SNAI3*; (downregulated): *BMP1, CDH2, CTNNB1, FN1, FTH1, FZD7, MELTF, MMP2, MMP9, MYC, PTGS2, SNAI2, TFRC, TWIST1, VIM, WNT5A, ZEB1,* and *ZEB2* (up to tenfold; *p* < 0.05; Figure 3A: volcano plot; Figure 3B: table)]. Strikingly, these genes modulated by *GLO1*_KO status encode various factors involved in EMT-like dynamics, as detailed in the discussion [13,25,26,27,28,29,30,31,32,33,34]. Taken together, these expression changes are consistent with EMT suppression as a result of *GLO1* deletion.

**Figure 4:**
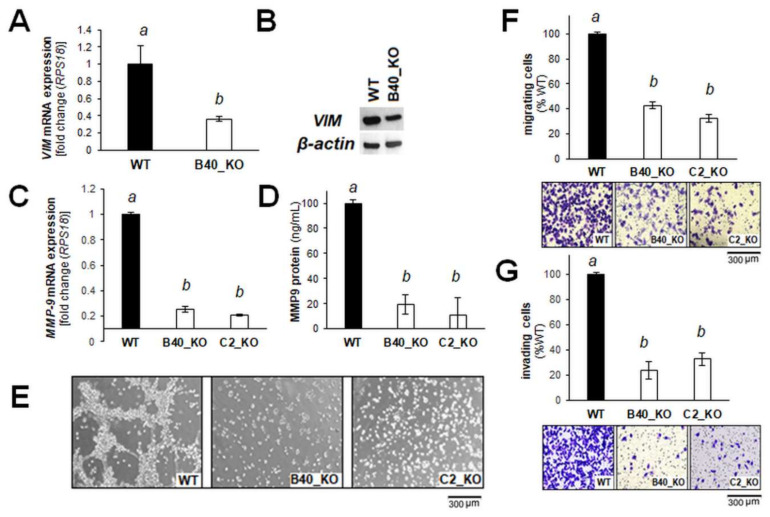
Genomic *GLO1* deletion impairs migration and invasion of A375 malignant melanoma cells. (**A**) RT-qPCR and (**B**) immunoblot assessment of vimentin expression (WT, B40_KO). (**C**) RT-qPCR and (**D**) ELISA assessment of MMP9 expression (WT, B40_KO, C2_KO). (**E**) Branching morphology, as assessed by light microscopy (20× magnification; WT, B40_KO, C2_KO; representative images). (**F**) Transwell migration (WT, B40_KO, C2_KO) and (**G**) invasion through Matrigel-coated Boyden chambers (WT, B40_KO, C2_KO); bar graphs depicted with representative images (after crystal violet staining of inserts.). The uncropped blots and molecular weight markers of Figure 4B are shown in Figure S3. For bar graph statistical analysis with letter designation see Materials and Methods Section 4.19.

Next, as representative examples relevant to a large number of cancers associated with EMT, we examined *VIM* and *MMP9* expression in more detail (assessed by individual RT-qPCR and immune-detection), which have also been examined in the context of EMT-related melanomagenesis [30,35,36,37]. Indeed, downregulation of *VIM* expression (encoding vimentin) in response to *GLO1* deletion was also confirmed by independent RT-qPCR and immunoblot analysis (Figure 4A,B). Likewise, further analysis confirmed attenuation of *MMP9* expression (detected by RT-qPCR and ELISA) as a function of *GLO1* deletion (Figure 4C,D). EMT-related changes (observable at the mRNA expression level) as a function of *GLO1* expression were also substantiated by phenotypic assessment performed in vitro (Figure 4E–G). At the cellular level, it was observed that A375-*GLO1*_KO (as compared to wildtype cells) display pronounced attenuation of branching morphology (Figure 4E), transwell migration (Figure 4F), and Matrigel 3D-invasion capacity (Figure 4G).

**Figure 5:**
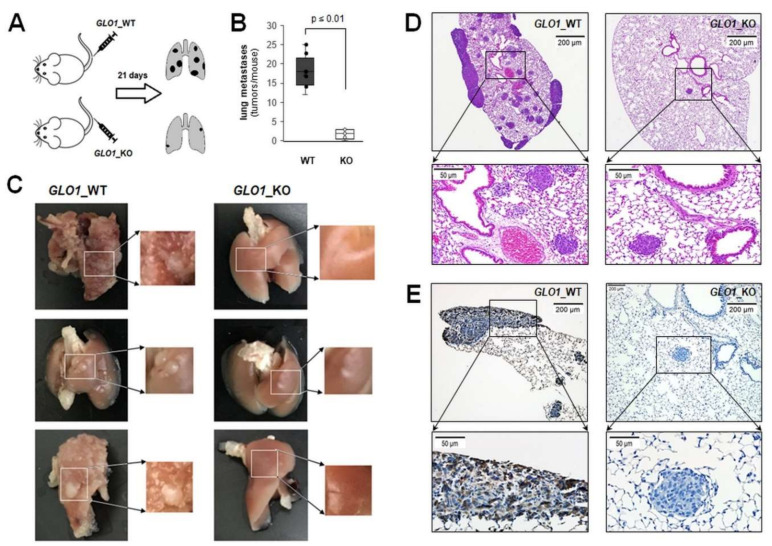
Genomic *GLO1* deletion impairs metastasis in a SCID mouse model of malignant melanoma. (**A**) Melanoma cells (WT, B40_KO) were tail vein injected (five mice per group) followed by analysis of lung metastasis 21 d later. (**B**) Box plot depicts numeric analysis of metastases per lung. (**C**) Total lung specimens were obtained upon harvest [with field magnification (right panel) for visualization and counting of metastases]; left panels: GLO1_WT; right panel: GLO1_KO. (**D**) Representative lung tissue cross sections were visualized by H&E staining or (**E**) GLO1 immunostaining, absent from GLO1_KO tumor tissue [taken at 4 × (top) versus 20 × (bottom) magnification].

Next, a SCID mouse metastasis model of human melanoma was employed assessing *GLO1*-modulation of lung tumorigenesis after tail vein injection of melanoma cells (Figure 5). To this end, three weeks after cell injection, lung tumor burden imposed by A375-*GLO1*_B40_KO versus A375-*GLO1*_WT cells was compared (Figure 5A). Upon necropsy, lungs from animals injected with A375-*GLO1*_WT displayed pronounced metastatic pathology, largely absent from tissue obtained from A375-*GLO1*_KO-injected mice, and tumor multiplicity was attenuated as a result of *GLO1* genetic deletion [3 ± 1 versus 18 ± 4 (tumors per lung; *p* < 0.001); (Figure 5B,C)]. Immunohistochemical analysis of lung tissue further substantiated reduced multiplicity and size of lung tumors in A375-*GLO1*_KO-injected mice (H&E; Figure 5D). As expected, these tumors displayed a striking absence of GLO1 epitopes (as evidenced by absence of brown staining employing IHC) as compared to tumors resulting from A375-*GLO1*_WT metastasis (Figure 5E), an observation consistent with absence of GLO1 protein levels in A375-*GLO1*_B40_KO cells examined by immunoblot analysis (Figure 1C). Interestingly, mouse necropsy also indicated metastasis into brain, heart, and intraperitoneal cavity observable in A375-*GLO1*_WT yet absent from tissue obtained from A375-*GLO1*_KO-injected mice, an observation awaiting further analysis (data not shown).

Taken together, these prototype data support a crucial mechanistic role of *GLO1* expression in melanomagenesis observable in vitro and in vivo.

### 2.3. Re-Expression of GLO1 in A375-GLO1_KO Cells Reverses EMT-Related Gene Expression and Restores Melanoma Cell Migration and Invasiveness In Vitro

**Figure 6:**
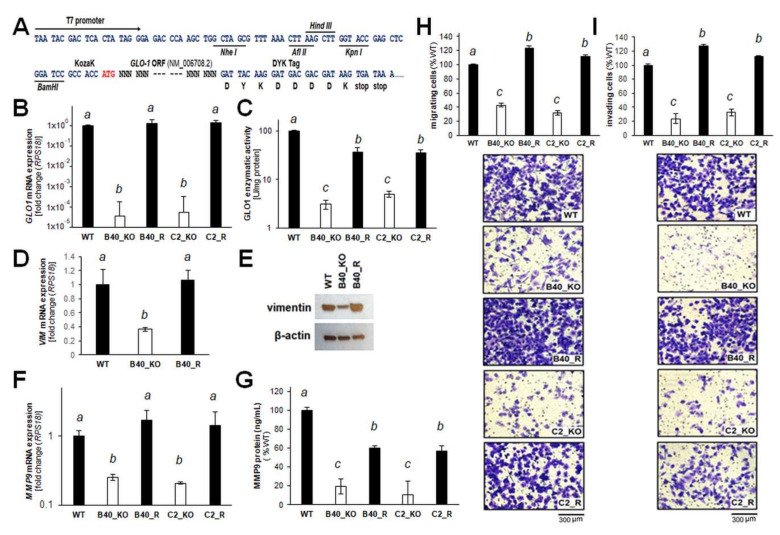
CMV-driven *GLO1* re-expression (A375-*GLO1*_R) restores the migration and invasion potential of A375 malignant melanoma cells with genomic *GLO1* deletion. (**A**) Insertion site of CMV-driven expression construct for stable transfection of A375-*GLO1*_KO clones. (**B**) RT-qPCR analysis of *GLO1* mRNA levels in A375 melanoma cell lines (WT, B40_KO, C2_KO, B40_R, and C2_R). (**C**) GLO1-specific enzymatic activity in A375 melanoma cell lines (WT, B40_KO, C2_KO, B40_R, and C2_R). (**D**) RT-qPCR and (**E**) immunoblot assessment of vimentin expression (WT, B40_KO, and B40_R). (**F**) RT-qPCR and (**G**) ELISA assessment of *MMP9* expression (WT, B40_KO, and B40_R). (**H**) Transwell migration (WT, B40_KO, C2_KO, B40_R, and C2_R) and (**I**) invasion though Matrigel-coated Boyden chambers (WT, B40_KO, C2_KO, B40_R, and C2_R). Bar graphs are depicted together with representative images after crystal violet staining of inserts. The uncropped blots and molecular weight markers of Figure 6E are shown in Figure S4. Letter designation has been specified according to respective section in Materials and Methods.

After demonstrating EMT impairment as a function of genetic *GLO1* deletion, further genetic evidence in support of a critical role of *GLO1* in melanoma invasion and metastasis was generated (Figure 6). First, a CMV-driven expression construct for stable transfection of A375-*GLO1*_KO clones was generated (Figure 6A). *GLO1* re-expression was confirmed in transfectants by RT-qPCR analysis of *GLO1* mRNA levels and detection of specific enzymatic activity, performed in A375 melanoma cell lines (WT, B40, C2, B40R, C2R; Figure 6B,C). Strikingly, RT-qPCR and immunoblot assessment of vimentin expression revealed a reversal of *GLO1*_KO-induced changes by *GLO1* expression rescue (WT, B40_KO, B40_R; Figure 6D,E). Likewise, re-expression of *GLO1* reversed downregulation of the EMT driver *MMP9,* as assessed by single RT-qPCR analysis and ELISA-based detection (Figure 6F,G). Using KO (KO_B40 and KO_C2) versus rescue clones (B40_R and C2_R)], it was then observed that an impaired transwell migration potential (characteristic of A375 malignant melanoma cells with *GLO1* deletion) can be fully restored by CMV-driven *GLO1* re-expression (Figure 6H). Likewise, an impaired potential for invasion, as assessed by Matrigel transwell assays (characteristic of A375 malignant melanoma cells with *GLO1* deletion), was fully reversed by CMV-driven *GLO1* re-expression (Figure 6I).

**Figure 7:**
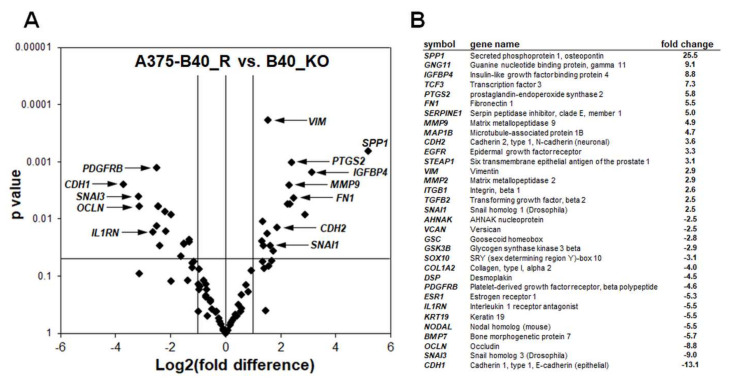
*GLO1* rescue (A375-*GLO1_*R) restores EMT-related gene expression in A375 malignant melanoma cells with genomic *GLO1* deletion. (**A**) Volcano plot depicting differential gene expression (B40_R versus B40_KO), as detected by the RT^2^ Profiler^TM^ PCR expression array technology (cut-off criteria: expression differential > 2; *p* ≤ 0.05; n = 3). (**B**) Numerical expression changes [B40_R versus B40_KO (*p* ≤ 0.05; n = 3)] revealing modulation of EMT-related genes as a function of *GLO1* expression and rescue.

Remarkably, EMT-directed gene expression array analysis, performed in analogy to our prior clonal characterization (Figure 3), confirmed that re-expression of *GLO1* reversed EMT-related mRNA expression changes characteristic of A375-*GLO1*_KO cells (Figure 7A: volcano plot; Figure 7B: table). For example, *MMP9*, downregulated in *GLO1*_KO clones [5.2-fold versus WT (Figure 3)] was now upregulated in A375-*GLO1*_R [4.9-fold versus A375-*GLO1*_KO (Figure 7)]. Likewise, *VIM*, *FN1*, *OCLN*, *SNAI3*, and numerous other EMT-related genes displayed a pattern of expression reversal [as labeled by arrows in the respective volcano plot (Figure 7A) and summarized numerically (Figure 7B)].

Taken together, these genetic rescue data provide strong experimental evidence supporting a novel role of *GLO1* expression as a critical molecular determinant of EMT-like characteristics in A375 malignant melanoma cells, observable at both the gene expression and phenotypic levels.

## 3. Discussion

Recently, we have reported the overexpression of glyoxalase 1 (encoded by *GLO1*), a glutathione-dependent enzyme involved in the detoxification of the reactive glycolytic byproduct methylglyoxal, in human malignant melanoma cell culture models and clinical samples [16]. In addition, in this study, phenotypic consequences of transient genetic knockdown (employing siRNA) were determined in various established and genetically diverse melanoma cell lines (including A375, G361, and LOX) that revealed sensitization to methylglyoxal-induced impairment of proliferation and viability as a result of genetic *GLO1* antagonism. Metabolic reprogramming is a molecular hallmark of cancer that has also been substantiated in melanoma. However, even though *GLO1* expression serves an essential function in the maintenance of glycolytic energy metabolism, the specific role of *GLO1* in melanomagenesis remains largely unexplored [2,5,22,38,39].

Here, using genetic target modulation, we have identified *GLO1* as a novel molecular determinant of invasion and metastasis in malignant melanoma. First, A375 human malignant melanoma cells with *GLO1* deletion (A375-*GLO1*-KO) were engineered using CRISPR/Cas9, and genetic rescue clones were generated by stable transfection of KO clones employing a CMV-driven *GLO1* construct (A375-*GLO1*-R) (Figure 1). After confirming *GLO1* target modulation at the mRNA and protein levels (RT-qPCR, immunodetection, enzymatic activity), phenotypic characterization indicated that deletion of *GLO1* does not impact proliferative capacity while causing significant sensitization to methylglyoxal-, chemotherapy-, and starvation-induced cytotoxic stress—all of which may result from impaired glycolytic flux downstream of *GLO1* deletion (Figure 2).

Strikingly, differential gene expression array analysis (A375-*GLO1*-KO versus A375-*GLO1*-WT) revealed pronounced modulation of EMT-related genes (Figure 3). These genes are encoding: (*I*) cellular adhesion factors, signaling molecules, and extracellular matrix components (including *BMP1, CTNNB1, CDH1, FN1, FZD7, IL1RN, CDH2, VIM, WNT5A*); (*II*) enzymes involved in extracellular matrix remodeling (including *MMP2, MMP9*) and inflammation (*PTGS2*); (*III*) transcription factors (including *MYC, SNAI2, SNAI3, TWIST1, ZEB1, ZEB2*); (*IV*) iron-regulatory factors (including *FTH1, MELTF, TFRC*)—all of which have been shown earlier to control cellular EMT and invasiveness in various tumor types [13,25,26,27,28,29,30,31,32,33,34,35,36,37].

EMT is now an established molecular pathway involved in melanoma progression, recognized also as an important therapeutic target, and our array analysis identified a number of EMT-regulatory genes responsive to *GLO1* status implicated mechanistically in metastatic melanoma [13,25,26,27,28,29,30,31,32,33,34]. The fact that these gene expression changes (associated with attenuation of EMT-potential) occur as a direct result of *GLO1* deletion was further substantiated by the fact that *GLO1* re-expression (A375-*GLO1*-R versus A375-*GLO1*-KO) largely reversed this expression pattern (Figure 7). For example, downregulation of EMT driver genes (A375-*GLO1*-KO versus A375-*GLO1*-WT) [such as *FN1* (3.2-fold), *MELTF* (2.5-fold), *MMP2* (2.8-fold), *MMP9* (5.2-fold), *MYC* (3.9-fold), *PTGS2* (7.4-fold), *SNAI2* (4.1-fold), *TFRC* (9.1-fold), *VIM* (2.7), *ZEB2* (3.3-fold)] was reversed by *GLO1* re-expression (A375-*GLO1*-R versus A375-*GLO1*-KO) [causing upregulation of *FN1* (5.5-fold), *MELTF* (2.5-fold), *MMP2* (2.9-fold), *MMP9* (4.9-fold), *MYC* (3.4-fold), *PTGS2* (5.8-fold), *SNAI1* (2.5-fold), *TFRC* (13.9-fold), *VIM* (2.9), *ZEB2* (2.2-fold)]. Likewise, upregulation of EMT-antagonistic genes as observed in *GLO1* KO cells (A375-*GLO1*-KO versus A375-*GLO1*-WT) [including *CDH1 (5.2)*, *IL1RN (3.9)*, *OCLN* (6.4-fold), *SNAI3 (5.4)*] was reversed by *GLO1* re-expression (A375-*GLO1*-R versus A375-*GLO1*-KO) [causing downregulation of *CDH1 (13.1)*, *IL1RN (5.5)*, *OCLN* (8.8-fold), *SNAI3 (9.0)*] [13,25,26,27,28,29,30,31,32,33,34]. Remarkably, even though CMV-driven forced expression of *GLO1* in rescue clones caused restoration of mRNA levels (Figure 6B), enzymatic activity was only moderately restored (Figure 6C), a differential expression readout that might be the result of ectopic expression associated with dysregulated levels of mRNA that are then not efficiently processed or translated. Likewise, it was observed that pronounced (approximately 25-fold) upregulation of *SPP1* encoding osteopontin, a secreted integrin-binding protein and established molecular prognostic marker for melanoma, occurred in response to *GLO1* rescue expression, whereas *SPP1* downregulation was not observed in *GLO1*_KO as compared to wildtype cells, again indicating a mechanistic discrepancy that might be related to the nature of CMV-driven forced rescue expression [40].

Providing further evidence in support of *GLO1* expression as a mechanistic determinant of melanoma cell invasiveness, differential expression of *MMP9* as a function of *GLO1* status was substantiated by enzymatic activity and ELISA analysis (Figure 4). Strikingly, phenotypic assessment revealed the pronounced attenuation of morphological potential, transwell migration, and Matrigel 3D-invasion capacity displayed by A375-*GLO1*-KO cells, reversed again in genetic rescue clones. Finally, using a SCID mouse metastasis model, it was observed that lung tumor burden imposed by A375-*GLO1*-KO cells was strongly attenuated as compared to A375-*GLO1-*WT cells (Figure 5). Taken together, these data provide strong genetic evidence in support of a novel function of *GLO1* in melanoma cell invasiveness and metastasis, and our ongoing investigations explore the function and therapeutic potential of *GLO1* as a novel molecular target in melanoma metastasis.

Interestingly, a mechanistic role of *GLO1* expression in cancer cell EMT has been suggested before; specifically, it has been demonstrated that *GLO1* sustains the metastatic phenotype of prostate cancer cells via EMT control [17,18,41]. Employing siRNA-based partial target modulation in DU145 and PC3 prostate carcinoma cells, inhibition of EMT-related gene expression (with downregulation of *MMP9*, *MMP2*, *VIM*, and *SNAI1,* among others) and suppression of migration and metastasis were assessed in vitro. Moreover, a role of the tumor suppressor miR-101 in the control of *GLO1* expression as a determinant of methylglyoxal-induced posttranslational modification of specific protein targets (including HSP40) was substantiated [18]. However, further experimental evidence will be needed to substantiate a similar pathway that might be operative in melanoma, and the specific mechanistic role of *GLO1* expression and enzymatic function (involving its oncometabolic substrate methylglyoxal) in the control of EMT-related gene expression remains unresolved at this point. Thus, it will be important to explore the potential role of methylglyoxal-dependent posttranslational adduction and modulation of specific molecular protein targets operating upstream of EMT and metastasis regulation. In this context, we are currently aiming at mass spectrometric identification of the methylglyoxal-adducted protein detected by us in *GLO1*_KO clones using an arg-pyrimidine antibody (Figure 1E).

Interestingly, a double-edged hormetic role of methylglyoxal in tumorigenesis is rapidly emerging, with moderately elevated intracellular concentrations enhancing proliferative and survival signaling through stress response pathway modulation, and high concentrations causing tumor cell apoptosis and blockade of tumorigenic progression [1,3,4,5,19]. Moreover, *GLO1* control of cancer stem cell expansion has been demonstrated, and recent evidence suggests a tumor-promoting role of methylglyoxal in cancer progression and EMT as observed in anaplastic thyroid, colorectal, and breast cancers [7,42].

Pharmacological inhibition of GLO1 enzymatic activity through employment of small-molecule inhibitors acting as glutathione antagonists has been explored before, and GLO1 is a promising target for the development of investigational therapeutics with anti-oncological and antimicrobial (e.g., *Trypanosoma cruzi* and *Leishmania*) activities [8,12,43]. Given the striking phenotypic effects observed by us in malignant cells with genetic *GLO1* deletion, it will be interesting to examine whether pharmacological target modulation (i.e., inhibition of enzymatic activity) phenocopies the changes caused by stringent genetic target modulation employing CRISPR/Cas9. Indeed, early studies indicate that GLO1 inhibition may show promise for chemosensitization of cancer cells, as substantiated further by the more recent identification of structure-based, non-glutathione competitive inhibitors with potent apoptogenic activity against cultured cancer cells [8,12,43].

It is important to note that our studies (employing CRISPR/Cas9-based generation of isogenic A375_*GLO1*_KO clones together with CMV-driven rescue clones) represent a limited yet powerful prototype approach towards a more stringent elucidation of *GLO1* function in melanomagenesis. Likewise, the use of isogenic NRAS mutant-A375 melanoma cell lines is firmly established, and the commercial availability of these lines enables the explorative molecular examination of genetic mechanisms underlying BRAF kinase inhibitor resistance and the BRAF RAS–RAF–MEK–ERK (MAPK) signaling pathway in melanoma [44]. However, more detailed follow up studies will have to examine the role of *GLO1* deletion in a genetically diverse set of representative human melanoma cell lines to further substantiate our prototype data.

Taken together, these data provide strong genetic evidence in support of a novel function of *GLO1* in melanoma cell invasiveness and metastasis, and ongoing investigations explore the function and therapeutic potential of *GLO1* as a novel oncological target and predictive marker in melanoma metastasis.

## 4. Materials and Methods

### 4.1. Chemicals

All chemicals and reagents were purchased from Sigma Chemical Co (St. Louis, MO, USA), unless specified otherwise.

### 4.2. Cell Culture

Human malignant A375 melanoma cells (CRL-1619; ATCC, Manassas, VA) and all engineered isogenic variants [CRISP/Cas9-derived *GLO1* KO cells (A375-*GLO1*_KO) and CMV-driven *GLO1* rescue cells (A375-*GLO1*_R)] were cultured in RPMI medium (Corning Inc., Corning, NY), supplemented with 10% FBS and 2 mM L-glutamine. Cells were maintained in a humidified incubator at 37 °C, 5% CO_2_ and 95% air.

### 4.3. CRISPR/Cas 9 Genome Editing

Homozygous knock-out of *GLO1* in human malignant A375 melanoma cells was generated using CRISPR/Cas9 technology at the University of Arizona Cancer Center (UACC) Genome Editing Facility. Briefly, double-strand breaks were produced on either side of *GLO1* exon 2 at the predicted sites on chromosome 6 at position 38,687,313 and 38,685,738 base pairs with guide CRISPR RNAs (crRNAs) corresponding to the sequences 5′-ACCCTCATGGACCAATCAGT-3′ and 5′-TGATCATAGGTGTATACGAG-3′, respectively. Parental A375 melanoma cells were transfected with Cas9 protein, crRNAs, and transactivating crRNA (Integrated DNA Technologies, San Diego, CA, USA) using the Lipofectamine RNAiMAX reagent (Thermo Fisher Scientific, Waltham, MA). Two days posttransfection, cutting efficiency was estimated based on DNA isolated from a portion of the transfected cell population using a T7 endonuclease assay (New England BioLabs, Ipswich, MA), with PCR primers (5′-ACAGTAGCAAGCATGGCAGT-3′ and 5′-GCACCAGTGAGGTTCACAGA-3′) flanking the predicted ligation junction product. Subsequently, single cells were deposited in 96-well plates (n = 10) by the UACC Flow Cytometry Shared Resource. Single-cell colonies were expanded and, after approximately three weeks, clones were screened by PCR and agarose gel electrophoresis. Clones that were negative for a sequence inside the targeted deletion (PCR primers 5′-TCACTCGTAGCATGGTCTGC-3′ and 5′-CTTTGGACTTGCATCACACAGG-3′) and negative for the undeleted chromosomal sequences but positive for ligation junction fragment were scored as potentially homozygous for *GLO1* exon 2 deletion. Absence of GLO1 protein was confirmed by immunoblot analysis and single RT-qPCR.

### 4.4. Rescue Expression Construct

GenEZ *GLO1* ORF clone (NM_006708.3; ATG GCA GAA CCG CAG CCC CCG TCC GGC GGC CTC ACG GAC GAG GCC GCC CTC AGT TGC TGC TCC GAC GCG GAC CCC AGT ACC AAG GAT TTT CTA TTG CAG CAG ACC ATG CTA CGA GTG AAG GAT CCT AAG AAG TCA CTG GAT TTT TAT ACT AGA GTT CTT GGA ATG ACG CTA ATC CAA AAA TGT GAT TTT CCC ATT ATG AAG TTT TCA CTC TAC TTC TTG GCT TAT GAG GAT AAA AAT GAC ATC CCT AAA GAA AAA GAT GAA AAA ATA GCC TGG GCG CTC TCC AGA AAA GCT ACA CTT GAG CTG ACA CAC AAT TGG GGC ACT GAA GAT GAT GAG ACC CAG AGT TAC CAC AAT GGC AAT TCA GAC CCT CGA GGA TTC GGT CAT ATT GGA ATT GCT GTT CCT GAT GTA TAC AGT GCT TGT AAA AGG TTT GAA GAA CTG GGA GTC AAA TTT GTG AAG AAA CCT GAT GAT GGTAAA ATG AAA GGC CTG GCA TTT ATT CAA GAT CCT GAT GGC TAC TGG ATT GAA ATT TTG AAT CCT AAC AAA ATG GCA ACC TTA ATG TAG) was cloned into the linearized pcDNA3.1 + /C-(K)DYK vector (5444 base pairs, containing Neomycin resistance gene for mammalian selection) using Seamless cloning technology (GenScript Biotech, Piscataway, NJ) used for stable transfection of human A375 melanoma cells. Briefly, stable A375-*GLO1* rescue cells (A375-*GLO1*_R) were generated by overnight incubation of A375-*GLO1*_KO cells with DNA (4 µg)-Lipofectamine^®^ 2000 (10 µg) complexes at 37 °C. After 24 h transfection, cells were passaged at 1:10 dilution into fresh growth medium. Selection antibiotic, neomycin (500 µg/mL), was added to the growth media 24 h later to select for single-cell colonies. Individual single-cell colonies were then expanded, and cells were tested for *GLO1* mRNA and protein expression.

### 4.5. RNA Extraction and Single Reverse Transcrition Quantitative Polymerase Chain Reaction (RT-qPCR)

Total RNA from A375 melanoma cells was isolated individually using Qiagen RNeasy Mini Kit (Qiagen Sciences, Gaithersburg, MD) according to the manufacturer’s protocol. RNA integrity was checked by the RNA 6000 Nano chip kit using Agilent 2100 Bioanalyzer (Agilent Technologies, Santa Clara, CA, USA). Human *GLO1* (Hs_02861567_m1, FAM), *GLO2* (Hs_00193422_m1, FAM), *MMP9* (Hs_00234579_m1; FAM) and *RPS18* (Hs_01375212_g1; VIC) 20X primer/probes were obtained from Thermo Fisher Scientific, Waltham, MA. Complementary DNA (cDNA) was synthesized from 500 ng of total RNA using master mix containing 10 × RT buffer, 5.5 mM MgCl_2_, 2 mM dNTPs, 2.5 μM random hexamers, 2 units of RNase Inhibitor and 62.5 units of Multiscribe Reverse Transcriptase. Reactions were performed in MJ Thermocycler PTC-200 (MJ Research, Watertown, MA) followed by these conditions: 10 min at 25 °C; 30 min at 48 °C and 5 min at 95 °C. Then, 10 ng cDNA was used to amplify human *GLO1*, *GLO2* and *MMP9* sequences. Conditions for quantitative PCR reactions were as follows: 10 min at 95 °C followed by 15 sec at 95 °C, 1 min at 60 °C for 40 cycles using ABI7500 Real-Time PCR System (Applied Biosystems, Foster City, CA, USA). PCR amplification of human housekeeping gene *RPS18* was used to control quality of the cDNA. Non-template controls were included on each PCR plate. *GLO1*, *GLO2* and *MMP9* expression levels were normalized to the *RPS18* control [ΔCt = Ct (gene of interest) – Ct (housekeeping gene)]. Amplification plots were generated and the Ct values (cycle number at which fluorescence reaches threshold) recorded.

### 4.6. Immunoblot Detection

After cellular protein extraction using RIPA buffer containing 50 mM Tris, pH 7.4, 150 mM NaCl, 1 mM EDTA, 1% Triton N-100, 1% sodium deoxycholate and 0.1% sodium dodecyl sulfate and protease inhibitor mixture (leupeptin, aprotinin, PMSF), equal amounts of total protein were separated using 4–15% SDS-PAGE gel (Bio-Rad laboratories, Irvine, CA, USA) transferred to PVDF membrane, and developed. The following antibodies were used: anti-GLO1 rabbit polyclonal (96032; Abcam, Cambridge, UK), anti-VIM rabbit polyclonal (13032; Santa Cruz, Dallas, TX, USA), anti-methylglyoxal mouse monoclonal [243074 (clone 9F11); Abcam, Cambridge, UK]. The following secondary antibodies were used: HRP-conjugated goat anti-rabbit antibody or HRP-conjugated goat anti-mouse antibody (Jackson ImmunoResearch Laboratories, West Grove, PA, USA). Membranes were incubated with ECL Western Blotting Detection Reagents (Amersham Biosciences, Piscataway, NJ) and exposed to BioMax XAR film (Kodak, Rochester, NY, USA). Equal protein loading was examined by β-actin detection using a mouse anti-actin monoclonal antibody (Sigma Aldrich, St. Louis, MO, USA).

### 4.7. Glyoxalase 1-Specific Enzymatic Activity

Glyoxalase 1-specific enzymatic activity in melanoma A375 cell cytosolic fractions was analyzed using a colorimetric glyoxalase 1 assay kit (241019; Abcam, Cambridge, UK) according to the manufacturer’s instructions [16]. Briefly, pelleted cells (~1–2 × 10^6^) were homogenized with 300 μL of ice-cold Glo I Assay Buffer containing protease inhibitor PMSF and centrifuged (12,000 g; 4 °C; 10 min). Supernatant cytosolic fractions were analyzed for protein content (Pierce™ BCA Protein Assay Kit, Thermo Fisher Scientific, Waltham, MA, USA); equal amounts (10 μg protein) were mixed with substrate and then examined for enzymatic activity by measuring absorbance at 240 nm in kinetic mode (room temperature; 10–20 min).

### 4.8. Flow Cytometric Analysis of Cell Viability

After treatment [methylglyoxal in medium (500 µm) or serum starvation in Hank’s Balanced salt solution (HBSS) containing 1 g/L glucose (24 h continuous exposure)], viability of A375-*GLO1*_WT and KO clones was assessed by annexinV-FITC/propidium iodide (PI) dual staining followed by flow cytometric analysis as published before [23,45,46]. Cells (100,000) were seeded on 35 mm dishes and received treatment 24 h later. Cells were then harvested 24 h later and stained using an apoptosis detection kit according to the manufacturer’s specifications (APOAF, Sigma Aldrich, St. Louis, MO, USA). Viable cells are located in the bottom left quadrant (annexinV-FITC^−^/PI^−^), whereas early apoptotic and late apoptotic/necrotic cells are located in the bottom right (annexinV-FITC^+^/PI^−^) and top right quadrants (annexinV-FITC^+^/PI^+^), respectively.

### 4.9. Cell Glo Viability Assay CellTiter-Glo^®^ Assay

The CellTiter-Glo Luminescent Cell Viability Assay (Promega; San Luis Obispo, CA, USA) was used to determine the number of viable cells based on quantitation of the ATP present which signals the presence of metabolically active cells [45,46]. Cells (5000/well) in 100 µL of culture medium were seeded in 96-well opaque-walled plate. Control wells containing medium without cells were included to obtain a value for background luminescence. Then, test compounds (dacarbazine and cisplatin) were added to experimental wells and incubated for 24 h following published standard procedures [24]. After incubation, the plate was equilibrated at room temperature for approximately 30 min and 100 µL of CellTiter-Glo Reagent was added to each well containing 100 µL of medium containing cells and mixed for 2 min on an orbital shaker to induce cell lysis. Then, plate was incubated at room temperature for additional 10 min to stabilize luminescent signal and luminescence was measured using BioTek Synergy 2 Reader (BioTek, Winooski, VT, USA).

### 4.10. Cell Proliferation Assay

A published standard procedure was followed [45,46]. Briefly, cells were seeded at 10,000 cells/dish on 35 mm dishes. After 24 h, cells were treated with test compound. Cell number at the time of compound addition and 72 h later was determined using a Z2 Analyzer (Beckman Coulter, Fullerton, CA, USA). Proliferation was compared with cells that received mock treatment.

### 4.11. Detection of Intracellular Oxidative Stress by Flow Cytometric Analysis

Induction of intracellular oxidative stress by MG was analyzed by flow cytometry using 2′,7′-dichlorodihydrofluorescein diacetate (DCFH-DA) as a sensitive non-fluorescent precursor dye according to a published standard procedure [45]. Cells were treated with MG (500 µm, 6 h), followed by DCFH-DA loading. Cells were incubated for 60 min in the dark (37 °C, 5% CO_2_) with culture medium containing DCFH-DA (5 µg/mL). Cells were then harvested and analyzed immediately by flow cytometry.

### 4.12. Determination of Reduced Cellular Glutathione Content

Intracellular reduced glutathione was measured using the GSH-Glo Glutathione assay kit (Promega; San Luis Obispo, CA, USA) [45,46]. Cells were seeded at 100,000 cells/dish on 35 mm dishes. After 24 h, cells were treated with test compound. At selected time points, cells were harvested by trypsinization and then counted using a Coulter counter. Cells were washed in PBS, and 10,000 cells/well (50 µL) were transferred onto a 96-well plate. GSH-Glo reagent (50 µL) containing luciferin-NT and glutathione-S-transferase was then added followed by 30 min incubation. After addition of luciferin detection reagent to each well (100 µL) and 15 min incubation, luminescence reading was performed using a BioTek Synergy 2 Reader (BioTek, Winooski, VT, USA). Data are normalized to GSH content in untreated cells.

### 4.13. Human RT^2^Profiler^TM^ PCR Expression Array

Total cellular RNA from A375 cells and their isogenic variants was isolated according to a standard procedure using the RNeasy Mini kit (Qiagen, Valencia, CA, USA). Reverse transcription was performed using the RT^2^ First Strand kit (Qiagen, Valencia, CA, USA) from 500 ng total RNA. For expression profiling, the RT^2^ PCR expression array technology (Qiagen, Valencia, CA, USA) was used as published before [45,46]. For EMT-related gene expression changes, Human Epithelial Mesenchymal Transition array was used. Quantitative PCR was run using the following conditions: 95 °C for 10 min, followed by 40 cycles of 95 °C for 15 s alternating with 60 °C for 1 min (Applied Biosystems, Carlsbad, CA, USA). Gene-specific products were normalized to a group of 5 housekeeping genes including *ACTB*, *B2M*, *GAPDH*, *HPRT1* and *RPLP0* and quantified using the comparative (ΔΔCt) Ct method as described in the ABI Prism 7500 sequence detection system user guide. Expression values were averaged across three independent array experiments, and standard deviation was calculated for graphing and statistical analysis as published before [45]. Volcano plot depiction displays each gene’s *p*-value and fold expression difference (log2 scale) with the selected covariate. Thus, statistically significant gene alterations occur at the top of the plot [(above the horizontal cut-off line (*p*-value threshold: 0.05)], and differentially expressed genes are depicted on either side (beyond the 2-fold cut-off line).

### 4.14. Human MMP9 Immunoassay

Quantikine MMP9 quantitative sandwich enzyme immunoassay ELISA (PDMP900; R&D Systems, Minneapolis, MN) was used to measure the 92 kDa Pro- and 82 kDa active forms in cell culture supernatants of parental A375 and their *GLO1* isogenic variants. Briefly, supernatants were collected and samples prepared by removing particulates by centrifugation. Then, 100 µL of standards, controls, and 100-fold diluted supernatant samples were added to 100 µL of assay diluent in MMP9 pre-coated wells followed by ELISA procedure as specified by the manufacturer. Colorimetric analysis (450 nm) was performed using a BioTek Synergy 2 Reader (BioTek, Winooski, VT). Results were normalized to total sample protein using the Pierce™ BCA Protein Assay Kit (Thermo Fisher Scientific, Waltham, MA, USA).

### 4.15. Transwell Migration/Invasion Assay

Either uncoated (migration) or Matrigel-coated (invasion) 8 μm pore size translucent 24-well plate transwell chambers (BD Biosciences, San Jose, CA, USA) were used to evaluate the migration and invasion potential of A375 cells following a published standard procedure [47]. Briefly, 600 μL of normal growth medium (10% FBS) was added to the bottom of each well and a total of 2.5 × 10^4^ cells resuspended in 250 μL of migration buffer (normal growth medium; 0.5% FBS; 0.1% BSA) were seeded on top. After 18 h incubation at 37 °C, 5% CO_2_, non-invading cells were removed by wiping the upper side of the membrane, and invading cells fixed with methanol and stained with crystal violet (Sigma-Aldrich, St. Louis, MO, USA). The number of migrating cells was quantified by counting 10 random fields per filter at 400× magnification.

### 4.16. Matrigel 3D Growth Assay (Branching Morphology Assay)

Following a published standard procedure, pre-warmed Matrigel (37 °C; 300 μL) was added to each well of a 24-well plate followed by solidification (2 h) in the biosafety cabinet [48]. Cells (20 × 10^4^; resuspended in 1 mL of normal growth medium) were then placed on top and incubated (37 °C, 5%CO_2_). After 24–48 h, light microscopy and data analysis were performed.

### 4.17. Metastasis Model In Vivo

A375 and its *GLO1*_KO isogenic variants (WT, *GLO1*_KO) were inoculated with 1 × 10^6^ cells resuspended in 100 μL HBSS using intravenous (IV) tail vein injection of SCID mice. The mice (n = 5 per group) were obtained from the University of Arizona Cancer Center SCID house colony at the age of 9 weeks with an average weight of 20 g. Mice were weighed before the beginning of the experiment and twice a week thereafter to check for signs of illness. When mice were looking moribund, a gross necropsy was performed and brain, lungs and liver were evaluated for presence and number of metastases. Photos and number of metastases were taken and tissues fixed in NBF. This study was performed in strict accordance with the recommendations in the Guide for the Care and Use of Laboratory Animals of the National Institutes of Health. The IACUC protocol was approved by the University of Arizona Institutional Animal Care and Use Committee (Mouse protocol number: IACUC 17–298).

### 4.18. Immunohistochemistry

After SCID mice from both groups were sacrificed, lung tissues were collected, fixed in NBF, processed and embedded in paraffin. Sections from each tissue block were counterstained with hematoxylin/eosin and investigated (by standard streptavidin biotin immunohistochemical technique) for the expression of GLO1 using anti-GLO1 monoclonal antibody (CPTC-GLO1-1; DSHB, Iowa City, IA, USA). Briefly, following deparaffinization and hydration, slides were washed and subjected to citric heated antigen retrieval (pH 6.0) for 20 min. Slides were then incubated with primary antibody. After overnight incubation, slides were washed and incubated with anti-mouse secondary antibody for 30 min, washed and incubated with the streptavidin/horseradish peroxidase for 30 min (RTU PK7200, Vector Laboratories, Burlingame, CA, USA). Then, slides were developed with a diaminobenzidine/hydrogen peroxide mixture for 4 min (Vectastain ABC, SK-4103, Vector Laboratories, Burlingame, CA, USA), counterstained with hematoxylin, dehydrated with graded alcohols and xylene, and mounted using a xylene based medium. A brown color indicated a positive stain. Negative controls were performed on each run, substituting the primary antibody with mouse IgG1 (X0931, Agilent/DAKO, Santa Clara, CA, USA).

### 4.19. Statistical Analysis

Unless stated differently, data sets were analyzed employing analysis of variance (ANOVA) with Tukey’s posthoc test using the Prism 4.0 software (Prism Software Corp., Irvine, CA, USA); in respective bar graphs, means without a common letter differ (*p* < 0.05). Experiments involved at least nine individual replicates per data point, except for gene expression array analysis performed with three independent biological replicates analyzed in triplicate format.

## 5. Conclusions

Using genetic target modulation, we report the identification of *GLO1* as a novel molecular determinant of invasion and metastasis in malignant melanoma. Given the striking phenotypic effects observed by us in malignant cells with genetic *GLO1* deletion, our ongoing investigations explore the function and therapeutic potential of *GLO1* as a novel molecular cancer target. If pharmacological target modulation phenocopies the changes caused by CRISPR/Cas9-based genetic target modulation, drug-like GLO1 inhibitors might emerge as a novel class of molecular therapeutics for the suppression of invasion and metastasis in malignant melanoma patients.

## Supplementary Materials

The following are available online at https://www.mdpi.com/2072-6694/12/6/1369/s1, Figure S1: The uncropped blots and molecular weight markers of Figure 1C, Figure S2: The uncropped blots and molecular weight markers of Figure 1E, Figure S3: The uncropped blots and molecular weight markers of Figure 4B, Figure S4: The uncropped blots and molecular weight markers of Figure 6E.
