# Supplementary material for: Genetic Target Modulation Employing CRISPR/Cas9 Identifies Glyoxalase 1 as a Novel Molecular Determinant of Invasion and Metastasis in A375 Human Malignant Melanoma Cells In Vitro and In Vivo
Source: Cancers (Basel). 2020 May 26;12(6):1369. doi: 10.3390/cancers12061369 (PMC7352620; doi:10.3390/cancers12061369)
Supplement: Supplementary file 1 [file cancers-12-01369-s001.pdf]

# Supplementary Materials: Genetic Target Modulation Employing CRISPR/Cas9 Identifies Glyoxalase 1 as a Novel Molecular Determinant of Invasion and Metastasis in A375 Human Malignant Melanoma Cells In Vitro and In Vivo

**Jana Jandova, Jessica Perer, Anh Hua, Jeremy A. Snell and Georg T. Wondrak**

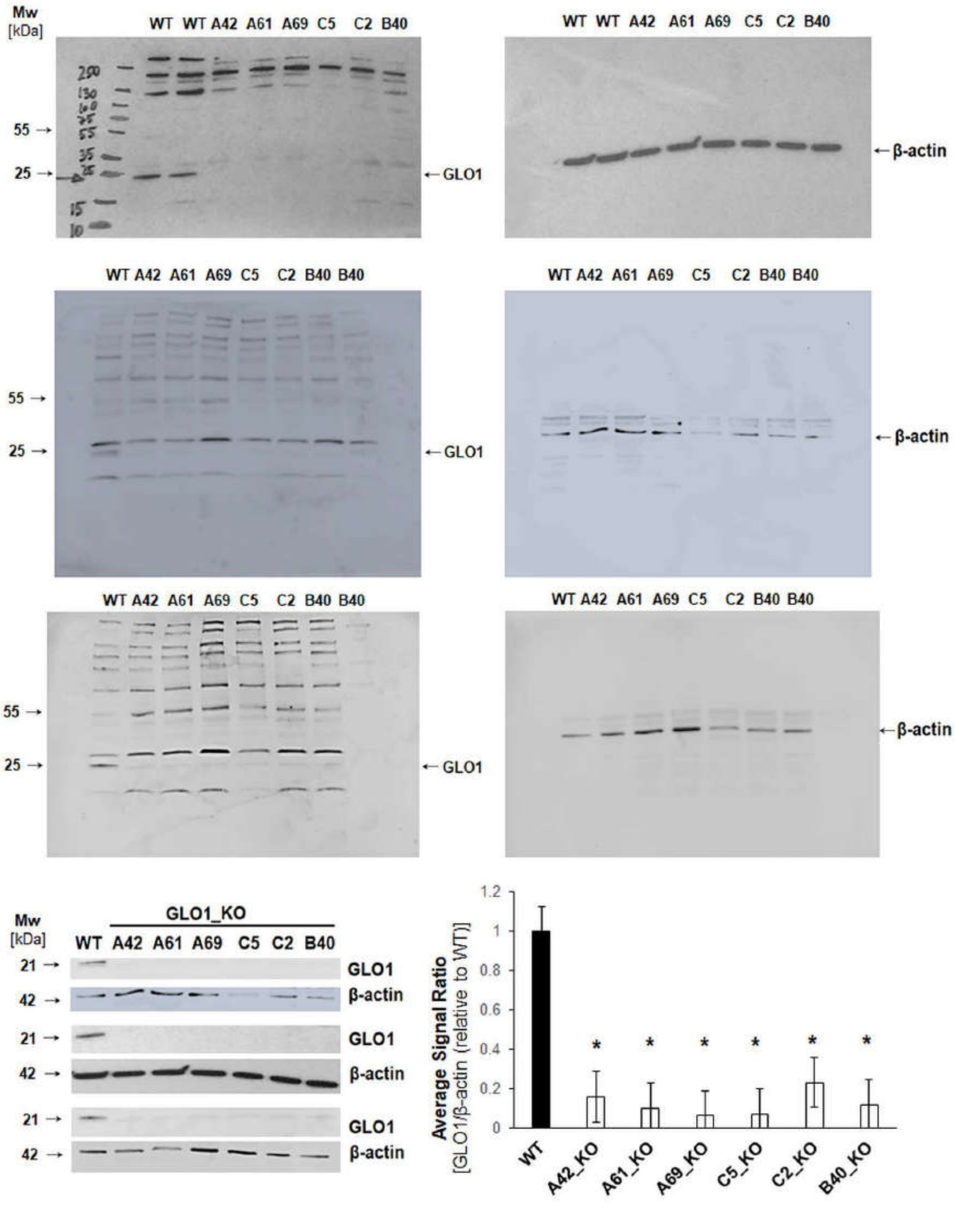


**Figure S1.** The uncropped blots and molecular weight markers of Figure 1C.

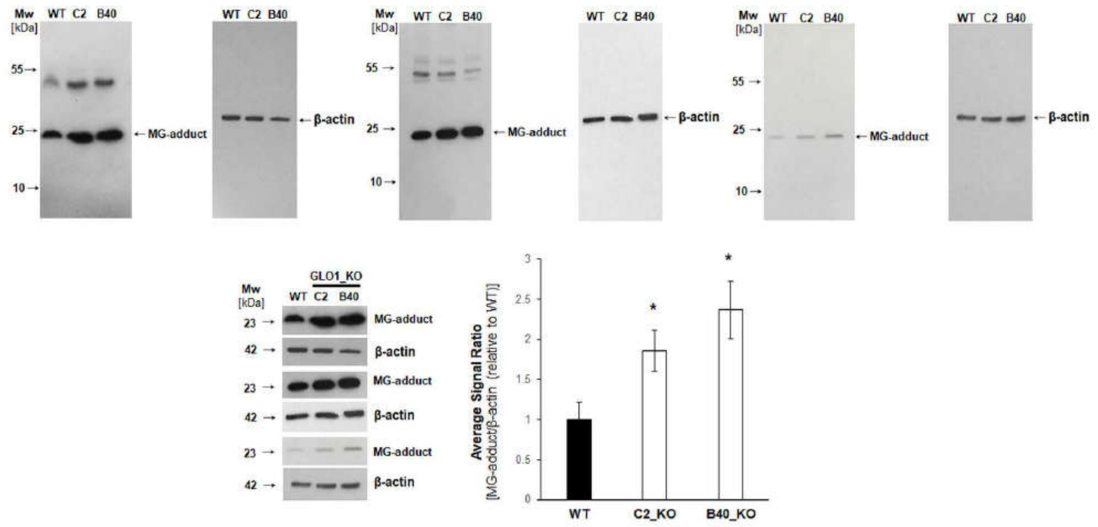


**Figure S2.** The uncropped blots and molecular weight markers of Figure 1E.

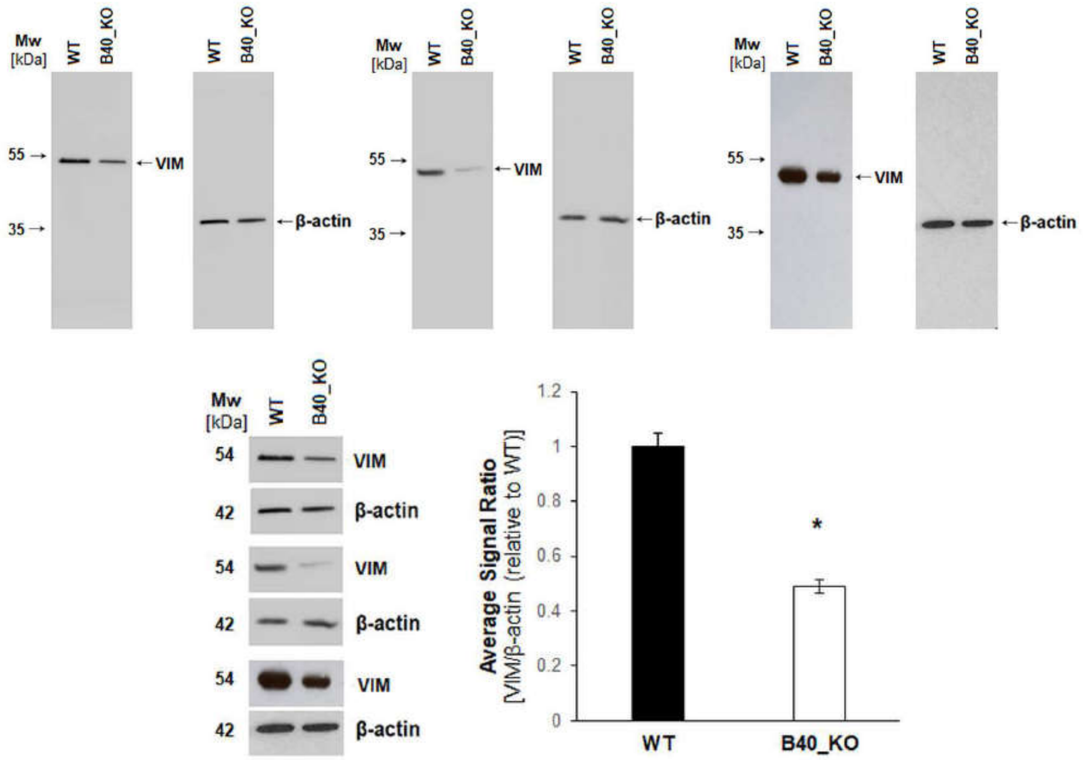


**Figure S3.** The uncropped blots and molecular weight markers of Figure 4B.

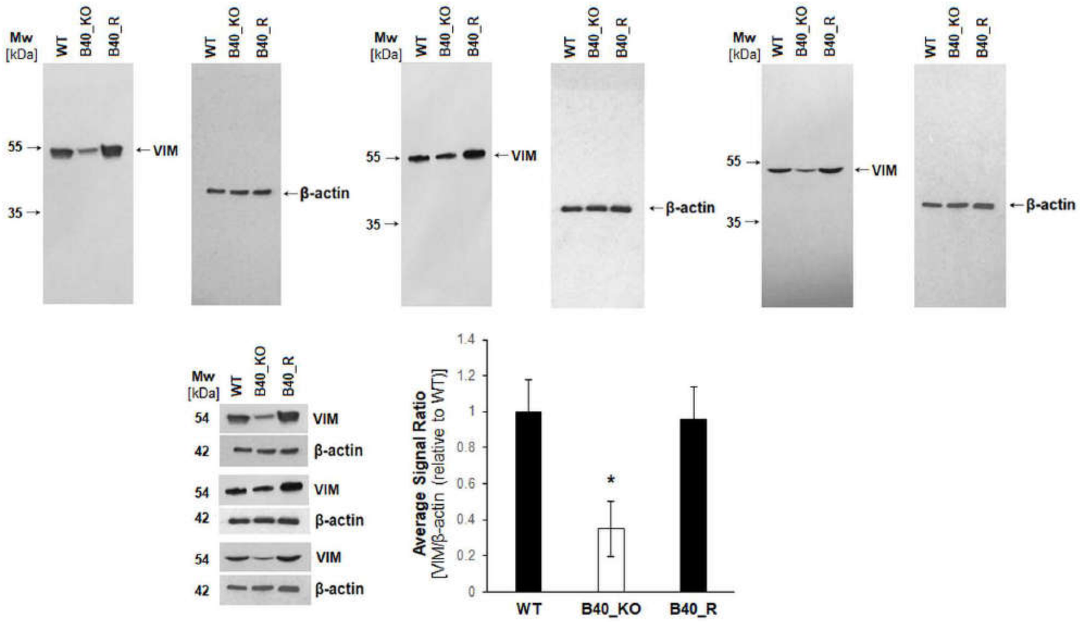


**Figure S4.** The uncropped blots and molecular weight markers of Figure 6E.

© 2020 by the authors. Licensee MDPI, Basel, Switzerland. This article is an open access article distributed under the terms and conditions of the Creative Commons Attribution (CC BY) license (http://creativecommons.org/licenses/by/4.0/).
